# Addressing the roadblocks to hypertension management in Ghana: proceedings of a roundtable discussion

**DOI:** 10.4314/gmj.v58i3.9

**Published:** 2024-09

**Authors:** Alfred Doku, Dzifa Ahadzi, Ebenezer A Adams, Aba A Folson, Elisa Codato, Francis Agyekum

**Affiliations:** 1 Department of Medicine, University of Ghana Medical School, Korle-Bu Teaching Hospital, Accra, Ghana; 2 Department of Internal Medicine, Tamale Teaching Hospital, Tamale, Ghana; 3 Stroke Association Support Network – Ghana, Accra, Ghana; 4 Department of Medicine, University of Health and Allied Sciences, Ho, Ghana; 5 World Heart Federation, Geneva, Switzerland

**Keywords:** Hypertension, roundtable, collaboration, stakeholders, Ghana

## Abstract

Hypertension is the top cause of preventable deaths in the African region. The burden of hypertension is increasing in Ghana, along with other countries in the African continent. Many people with hypertension in Ghana are unfortunately unaware of their diagnosis, and those who are aware often have poorly controlled blood pressure. The current situation is a signal of critical gaps in hypertension care in Ghana that need to be urgently addressed to curb this epidemic. The World Heart Federation (WHF), sponsored by Resolve to Save Lives, responded to this need by organising advocacy roundtables on hypertension in selected countries in sub-Saharan Africa to scale up hypertension management efforts in these countries. The roundtable on hypertension in Ghana was organised in collaboration with the Ghanaian Society of Cardiology (GSC) and the Stroke Association Support Network (SASNET) Ghana. A country mapping detailing the current situation and evolution of hypertension in Ghana over the years formed the basis of the roundtable discussions. The roundtable convened diverse stakeholders in hypertension care in Ghana to discuss the roadblocks to hypertension management in Ghana, proffer solutions to address them and chart a course with timelines for action. Major roadblocks to the management of hypertension in Ghana identified included inadequate financing for cardiovascular disease (CVD) care, inadequate capacity for CVD care across all levels of the healthcare system and insufficient education to empower people with hypertension for optimal self-care.

**Funding:**

The roundtable was funded through a grant by Resolve to Save Lives through the World Heart Federation.

## Introduction

Approximately 80 million adults above age 25 in Africa had hypertension in the year 2000, with a projected rise to 150 million by 2025.^1^ Hypertension is the main driver of stroke and heart disease, with dire outcomes of disability and premature death.^1^ Almost 90% of hypertension-related deaths occur in low- and middle-income countries, making hypertension the top cause of preventable death in these areas.^2^,^3^ Unfortunately, in their third to fourth decade of life, these devastating consequences affect Africa's young adults most.^1^ The alarming rate at which the burden of hypertension is rising in the African region is attributed to the epidemiologic transition with rapid urbanisation, weak health systems, limited availability and affordability of hypertension medications, and limited funding for non-communicable diseases (NCDs), including cardiovascular diseases (CVDs).^4^ many roadblocks need to be urgently addressed to curb the hypertension epidemic in Africa, and collaboration with relevant stakeholders is crucial to finding feasible and sustainable solutions.

## Methods

The World Heart Federation (WHF) responded to the urgent need to scale up efforts in hypertension management by organising advocacy roundtables in selected countries in sub-Saharan Africa, sponsored by a grant from Resolve to Save Lives. In July 2022, the Ghanaian Society of Cardiology (GSC) and Stroke Association Support Network (SASNET) Ghana were invited to collaborate with the WHF to organise a roundtable on hypertension in Ghana as part of this initiative. The Ghana Heart Initiative (GHI) co-funded the event and organised as a 2-day program from 31^st^ August to 1^st^ September 2022. The goal was to bring together a diverse group of key stakeholders to assess the roadblocks to managing hypertension in Ghana and identify context-appropriate solutions with timelines for action.

A country mapping concerning hypertension was conducted before the event to understand better the current situation and the evolution of hypertension over the years in Ghana from a multilevel perspective. The country mapping included data ranging from epidemiological trends, national policies and guidelines related to CVD and hypertension to data on the availability and accessibility of health services and hypertension treatment, among others. The data was collected by reviewing available literature on hypertension in Ghana, including scientific literature, official publications of the Ministry of Health (MOH), global databases, and websites of organisations working on NCDs in Ghana.

The country mapping results were shared with stakeholders before the meeting. The roundtable included clinicians, patient representatives, government representatives, local and international non-governmental organisations involved in hypertension care in Ghana, hypertension researchers, patient advocates, and the media.

The event included talks, plenary sessions, and brainstorming sessions. The areas emerging as foci for dialogue and brainstorming from the country-mapping results were financing and governance, physical and intellectual resources, and health delivery and information systems. These topics were assigned to three different groups, which were well-composed and included representatives of all cadres of participants present. Each group was tasked to summarise their key findings, including proposed solutions, timelines for action, and the key actors involved in delivering these solutions.

## Results

The country mapping revealed a national prevalence of hypertension of 34% among adults.^5^ Hypertension awareness was 49%, 36% were on treatment, and 18.6% were treated to target.^6^ The results showed that, despite the high burden of hypertension, there are several roadblocks to adequate control of hypertension in Ghana. Though there are over 1,700 health facilities in Ghana^7^, 73% of rural residential clusters do not have ready access to a nearby health facility.^8^ The rural population is 46 times more underserved than the urban population.^9^ Additionally, the cardiovascular workforce is woefully inadequate, with 20 cardiologists at the forefront by 2020.^10^ Concerning guidelines for the management of hypertension and its risk factors, the country mapping identified many local guidelines, including the recently launched Ghana National Guidelines for the Management of Cardiovascular Diseases (CVDs).^11^ Though the national guideline mentions fixed-dose combinations, they are not included in the essential medicines list. Furthermore, hypertension medicines seem generally unaffordable, and some patients face significant barriers to accessing prescribed medications.^12^ Additionally, Ghana lacks guidelines for task-sharing and task-shifting, simplified hypertension protocols, and national policies for CVD management.

Key international and local stakeholders attended the roundtable. The WHF president opened the meeting with a presentation on the organisation's key hypertension activities, focusing on collaboration opportunities with its members to support hypertension management locally. An example of the result of these collaborations is the WHF and Pan-African Society of Cardiology (PASCAR) updated Roadmaps for hypertension, presented by a representative of PASCAR. This presentation highlighted how these roadmaps can be adopted and used nationally in Ghana by drawing from the Kenyan experience. Representatives of the MOH and Ghana Health Service (GHS) gave brief addresses highlighting the timely nature of the roundtable as a useful forum to identify the roadblocks to hypertension management in Ghana and recommend actionable solutions.

A representative from the World Health Organisation (WHO) Headquarters presented the WHO guidelines on the pharmacological treatment of hypertension in adults and the HEARTS Technical Package. In contrast, a WHO Ghana office representative discussed the Package of Essential NCD Interventions (PEN package) as a tool for supporting NCD management in low-resource settings. An executive of the GSC discussed the National Guidelines for the Management of CVDs and its utility in hypertension management in Ghana.

Partner agencies involved in hypertension interventions in Ghana made presentations highlighting their unique strategies and results. The GHI expounded on their key role in the publication of the National Guidelines for the management of CVD with an accompanying mobile app version (Akomacare), the establishment of a national CVD support and call centre, the development of training manuals with training programs, and equipment support for health professionals nationally.

The Program for Appropriate Technology in Health (PATH) shared on the Happy Heart Africa project, a partnership with GHS and Astrazeneca aimed at providing sustainable measures to strengthen and support the local health systems through training and guidelines in hypertension management. With concrete examples from the Akomapa project (a collaboration with a Christian Health Association of Ghana (CHAG) district hospital, Novartis and the German Agency for International Cooperation (GIZ)) and the ADHINCRA (Addressing Hypertension Care in Africa) program in Ghana, Medtronic Labs discussed the use of technology-powered healthcare delivery models to manage hypertension. Representatives of the Task-Strengthening Strategy for hypertension control (TASSH) study discussed lessons learnt from implementing a health systems strengthening initiative to improve hypertension care at the community level. Novartis highlighted their innovative models to increase hypertension awareness through education and screening activities in collaboration with SASNET-Ghana and Life from 30. The Ghana NCD Alliance, Pharmacy Council of Ghana, National Health Insurance Scheme, physicians from various levels of the healthcare system, patient representatives and the media were present and contributed significantly to the discussions.

## Discussion

### Financing and Governance

Inadequate financing for CVDs was identified as the main roadblock to financing and governance (Table 1). Governmental funding for CVDs was suggested to be augmented by exploring domestic funding avenues. Suggested funding opportunities include increasing sin taxes (eg, smoking tax), encouraging corporate body funding as part of corporate social responsibility, and allocating a percentage of the proceeds from Ghana's natural resources to CVD management. These solutions would require the combined effort of key actors like the MOH, GHS, GHI, Ghana Revenue Authority, Parliament, and natural resource industries. For sustainable and accessible nationwide preventive CVD care, screening activities across all levels of care should be reimbursed by the NHIA. In addition, a bag pack containing the basic tools for CVD screening, including a blood pressure measuring device, glucometer, weighing scale, and a simplified CVD management protocol, should be provided at the community level for CVD screening activities.

**Table 1:** Major Roadblocks to the Management of Hypertension in Ghana

Roadblock	Solutions	Timelines	Actors to be involved
**Financing and governance**			
**Funding for NCDs and CVDs is inadequate.**	Engage NHIA to consider reimbursement of preventive services across the various levels of care	Short term (12 months)	NHIA
	Explore domestic avenues for funding of CVDs and NCDs including: Increase of sin tax Increase taxes on tobacco, alcohol and sugar-sweetened beverages. Corporate social responsibility Advocate for a percentage from proceeds from natural resources and oil companies to be allocated for CVDs and NCDs	Medium to long-term (1 – 5 years)	MOH GHS Mining companies MOH GHI NHIA GRA Local Organisations such as religious-based organisations
**Screening and diagnostic materials and services are not available (BP devices, screening equipment, weighing scales, glucometer etc.)**	Ministry of Health and GHS to work together with external donors and partners in providing the needed logistics (e.g. World Bank, NGOs, Pharma companies) Engage NHIA to consider reimbursement for preventive services across the various levels of care	Short term	MOH GHS District Assembly Health Funds Donor partners Government sources GNPC
	Specific funding allocated to cover cost of screening material for CVD/NCD, including of recalibration and maintenance of equipment Change the current policy to allow remuneration of specialist outreaches and screening by NHIA.	Medium to long term	
**Physical and intellectual resources**			
**Fixed dose combination for hypertension not included in the Essential Medicines List**	Engage the MOH/GHS to consider adding FDC and other hypertension medicines to the essential medicines list Include FDC in the Standard Treatment Guidelines during the next review, with the aim of making it available on NHIA.	Short term	MOH/GHS NHIA GNDP
**Shortage of human resources for health to address CVDs**	The curriculum should be revised to include basic training on CVDs at CHOs training institutions. Compulsory update courses for CPDs and health workers in the periphery to build capacity. Leverage on community volunteers and community nurses to do follow-ups of patients.	Short to medium-term	MOH/GHS MOE Professional bodies and associations Development partners
	Recruit more physician assistants and non-physician health workers, train them, and equip them to do some level of biochemistry screening. Train physician assistants with basic knowledge of hypertension care. Schedule specialist visits to help build their capacity. Develop a policy document to allow physician assistants to visit various CHPS zones with appropriate antihypertensive medication and onward distribution by the CHOs to specific patients.	Medium term	
**Healthcare delivery and information systems**			
**Patients are not adequately educated and receive misleading information from herbalists, traditional care givers, religious actors which affect compliance to treatment**	Preparing audio-visual tools for communication and education in English and local languages (TV/Radio messages and educational leaflets). Outreaches to local churches and mosques, involving the private sector and local pharmacies in correcting false beliefs. Establish patient-centred care and strengthen healthcare workers' education.	Short term	GHS, SASNET, Media Houses Clinicians and care providers MOH and FDA
	Enforce laws regulating herbal medications and monitor adverts,	Medium to long term	
**Fragmentation of Electronic Medical Records (EMR) systems leading to human error, delay in diagnosis and losses to follow up.**	Develop policies to harmonise and integrate EMR systems to allow data to be captured and shared at all levels of care. Develop automated alerts coded into EMR (e.g. when BP is elevated at two takes, send reminders of appointment)	Medium to long term	MOH EMR Systems managers at GHS Teaching hospitals CHAG & Private health facilities

GRA – Ghana Revenue Authority, GNPC – Ghana National Petroleum Company, GNDP – Ghana National Drug Policy, MOE – Ministry of Education, Short term: 12 months, Medium term: 1 to 3 years, Medium to long term: 1 to 5 years

### Physical and intellectual resources

Considering physical and intellectual resources, inadequate capacity for CVD care was considered a major roadblock. A suggested short-term measure was to engage the MOH, GHS, Ministry of Education and relevant regulatory professional bodies to revise the curriculum of community health nurses to include basic training on CVDs. In addition, continuous professional development in CVD care should be mandatory at all levels of the healthcare system. Coaching visits from higher cadre CVD professionals should be considered.

### Health Delivery and Information Systems

Inadequate patient education as a component of healthcare delivery and information systems was identified as a major roadblock to hypertension management. The problem is compounded by the miseducation of patients by some alternate care providers, such as herbalists, traditional caregivers, and religious bodies, leading to poor compliance with prescribed treatment. To mitigate this, key stakeholders such as the GHS, SASNET-Ghana, and media houses should collaborate to develop audio-visual educational materials for patients in English and local dialects. Healthcare workers should also be encouraged to use every clinic visit to address pertinent questions of patients with hypertension. Lastly, herbalists and traditional caregivers should be appropriately regulated by relevant agencies such as the Food and Drug Authority of Ghana.

## Outcome

WHF, GSC and SASNET-Ghana representatives convened a smaller committee of key stakeholders to discuss the results following the roundtable. The objective was to identify priority areas that would be easy to implement and to outline strategies for action as a country.

Progress has been made in forming a CVD Technical Working Group under the auspices of Ghana Health Services. This group comprises multidisciplinary CVD experts and stakeholders across Ghana, whose mandate includes overseeing the implementation of some of the actionable solutions suggested at the roundtable. Patient and healthcare worker education has been championed through the Ghanaian Society of Hypertension's annual May Hypertension Awareness campaign since 2023. The GHI has collaborated with GHS to create an online CVD course for healthcare workers, piloted in April 2024, including a hypertension care module.

## Limitations

Despite providing viable solutions to address the roadblocks to hypertension care in Ghana, a few limitations were observed. The country-mapping document that formed the basis of the roundtable discussion was based on online literature - official documents from governmental and global databases and informal data sources, including websites of different organisations working on hypertension in Ghana and press releases. There were no clear inclusion and exclusion criteria, and the data was not subjected to rigorous scientific evaluation as per a standard systematic review. Hence, the information provided was largely based on secondary data, lacking robust data on associated CVD risk factors, hypertension-mediated target organ damage, and patient perspective studies, which could have further enriched the discussions.

## Conclusion

The roundtable was a unique opportunity to convene key stakeholders in hypertension care in Ghana to escalate hypertension control efforts. Using a multi-perspective approach to problem-solving, many valuable lessons were learned. These lessons have formed the basis of meaningful interventions in hypertension care in Ghana, with the ultimate goal of curbing the rising prevalence of hypertension in Ghana.
